# Germline Mutation in *RNASEL* Predicts Increased Risk of Head and Neck, Uterine Cervix and Breast Cancer

**DOI:** 10.1371/journal.pone.0002492

**Published:** 2008-06-25

**Authors:** Bo Eskerod Madsen, Eliana Marisa Ramos, Mathieu Boulard, Katarzyna Duda, Jens Overgaard, Marianne Nordsmark, Carsten Wiuf, Lise Lotte Hansen

**Affiliations:** 1 Institute of Human Genetics, University of Aarhus, Aarhus, Denmark; 2 Bioinformatics Research Center (BiRC), University of Aarhus, Aarhus, Denmark; 3 Department of Experimental Clinical Oncology, University Hospital of Aarhus, Aarhus, Denmark; Peninsula Medical School, United Kingdom

## Abstract

**The Background:**

Ribonuclease L (*RNASEL*), encoding the 2′-5′-oligoadenylate (2-5A)-dependent RNase L, is a key enzyme in the interferon induced antiviral and anti-proliferate pathway. Mutations in *RNASEL* segregate with the disease in prostate cancer families and specific genotypes are associated with an increased risk of prostate cancer.

Infection by human papillomavirus (HPV) is the major risk factor for uterine cervix cancer and for a subset of head and neck squamous cell carcinomas (HNSCC). HPV, Epstein Barr virus (EBV) and sequences from mouse mammary tumor virus (MMTV) have been detected in breast tumors, and the presence of integrated SV40 T/t antigen in breast carcinomas correlates with an aggressive phenotype and poor prognosis.

A genetic predisposition could explain why some viral infections persist and induce cancer, while others disappear spontaneously. This points at *RNASEL* as a strong susceptibility gene.

**Methodology/Principal Findings:**

To evaluate the implication of an abnormal activity of RNase L in the onset and development of viral induced cancers, the study was initiated by searching for germline mutations in patients diagnosed with uterine cervix cancer. The rationale behind is that close to 100% of the cervix cancer patients have a persistent HPV infection, and if a defective RNase L were responsible for the lack of ability to clear the HPV infection, we would expect to find a wide spectrum of mutations in these patients, leading to a decreased RNase L activity. The HPV genotype was established in tumor DNA from 42 patients diagnosed with carcinoma of the uterine cervix and somatic tissue from these patients was analyzed for mutations by direct sequencing of all coding and regulatory regions of *RNASEL*. Fifteen mutations, including still uncharacterized, were identified. The genotype frequencies of selected single nucleotide polymorphisms (SNPs) established in the cervix cancer patients were compared between 382 patients with head and neck squamous cell carcinomas (HNSCC), 199 patients with primary unilateral breast cancer and 502 healthy Danish control individuals. We found that the genotype frequencies of only one of the 15 mutations, the yet uncharacterized 5′UTR mutation rs3738579 differed significantly between cancer patients and control individuals (P-value: 4.43×10^−5^).

**Conclusion/Significance:**

In conclusion, we have discovered an increased risk, a heterozygous advantage and thereby a protective effect linked to the *RNASEL* SNP rs3738579. This effect is found for patients diagnosed with carcinoma of the uterine cervix, HNSCC, and breast cancer thus pointing at *RNASEL* as a general marker for cancer risk and not restricted to familial prostate cancer.

## Introduction

Ribonuclease L (*RNASEL*) encoding the versatile endoribonuclease RNase L is a key enzyme in the interferon induced antiviral and anti-proliferate pathway, involved in cellular viral defense, single-stranded RNA cleavage and tumor suppressor activities as stress mediated apoptosis, cell proliferation and regulation of protein synthesis [1]–[5].

RNase L is activated in response to viral infection and the presence of virally encoded dsRNA mobilizes the type I interferons (IFN), which activate a family of 2′-5′ oligoadenylate synthetases (OAS) [6], [7]. With ATP as substrate these enzymes produce 5′-phosphorylated 2′-5′ linked oligoadenylates (2-5A), which activate RNase L by binding to the ankyrin repeats and inducing dimerization, essential for the endonuclease activity of RNase L [8].

Human RNase L comprises 741 amino acids, and three different domains, a nine ankyrin repeat in the N-terminal, a kinase-like and an RNase domain in the C-terminal part. The ankyrin repeats are known to be involved in protein/protein interactions, but the RNase L ankyrin repeats (2 to 4) bind the 2-5A molecule, which initiates the conformational change of RNase L leading to dimerization and thereby activation of the enzyme [9], [10]. Based upon sequence comparison it is predicted that RNase L should have protein kinase activity, but it has not been experimentally verified [11]. However, two missense mutations (R462Q and K392R) located in the protein kinase region have been shown to affect the enzyme dimerization and activity [12]. R462Q is a frequent and well-characterized mutation and homozygosity for the rare allele reduces the activity of RNase L by 3 fold [13]. K392R, located in the protein kinase-like region II and implicated in binding of ATP in kinases, prevents the dimerization of RNase L and thereby the activation of the enzyme [12]. The kinase-like and ribonuclease domains are related to those found in IRE 1, a transmembrane serine/threonine protein kinase receptor involved in the response to unfolded proteins in the ER [14].

*RNASEL* is located within the hereditary prostate cancer 1 (HPC1) region at 1q25.3. Chromosomal gain comprising this region has been found as a frequent event in uterine cervix cancer [15] and HNSCC [16] and amplification of the entire chromosome arm 1q is considered an early event in breast carcinogenesis [17].

Germline mutations in *RNASEL* segregate with the disease in prostate cancer families with linkage to the HPC1 region at 1q25.3 [18]. The majority of missense mutations are found within exon 2 encoding the ankyrin repeats and part of the kinase-like domain. Cells from carriers of M1I and E265X showed half the normal activity of RNase L and the normal allele was lost in tumor cells from patients heterozygous for these muations [18]. E265X terminates translation within the 2-5A binding domain of RNase L, a similar mutation has been shown to eliminate 2-5A binding in mice [1]. The mutation was originally identified in four brothers, three of which suffered from aggressive prostate cancer [18]. Carriers of E265X develop prostate cancer on an average of 11 years before non-carriers from the same families [19]. A founder mutation 471delAAAG, resulting in a truncated protein, is associated with prostate cancer in Ashkenazi Jews [20]. A number of missense mutations are found in hereditary prostate cancer (HPC) families as: G59S, I97L, I220V, S406F, R462Q, Y529C and D541E, no mis- or nonsense mutations have been found in the ribonuclease domain. The mutations G59S, I97L, I220V, G296V, S322F, Y529C and D541E showed normal level of RNase L when measured in a mouse RNase L^−/−^ cell line [3].

Due to the tumor suppressor activities of *RNASEL* it is suggested that RNase L directly or indirectly suppress one or more steps in the prostate tumorigenesis or metastasis formation [21]. Germline mutations in *RNASEL* have been intensively studied in sporadic and familial prostate cancer but the results are contradictive. Recently, a comprehensive meta-analysis comprising the mutations D541E, R462Q and E265X concluded that the genotype E541 increased the risk of developing prostate cancer for Caucasian men, regardless of a family history of the disease [21]. In contrast, E541 was found to increase the risk of prostate cancer in Japanese families with multiple affected members [22]. A marginal effect has been seen for E541 in the Swedish population but studies on other populations could not confirm these results [13], [19], [23], [24].

R462Q is located in the kinase-like domain, and the R462Q variant is capable of binding 2-5A, with a reduced ability to dimerize, a configuration necessary for the enzymatic activity [3]. The catalytic activity of the mutant enzyme is decreased three fold when compared to the wild type RNase L, and it is no longer capable of inducing apoptosis [13]. Males heterozygous for R462Q have a 1.5-fold elevated risk, and homozygous men double the risk of developing prostate cancer, suggesting R462Q to be a predictive marker for the malignancy [13].

Association between the R462Q carriers and risk of developing prostate cancer seems to depend highly on ethnicity. Mutations in *RNASEL* are found at a very low rate in the German and Swedish population, and no significant association was found between R462Q and risk of prostate cancer [23], [25]. Homozygosity for 462Q was significantly more frequent in Finnish prostate cancer families than in controls, and in Euro-Americans with a family history of prostate cancer, R462Q was inversely associated with low grade and low stage disease [19], [26]. A significant association was found between R462Q and an increased risk of early-stage and low-grade disease in Euro-Americans patients with no family history [26]. In Afro-Americans R462Q was associated with a positive family history and high-grade tumors [26].

Patients with a familial pancreatic cancer, which are homozygous for 462Q, presented a more aggressive disease with a high risk of developing metastasis [27] and the genotype is associated with age of onset of hereditary non-polyposis colon cancer (HNPCC) [28]. No association was found between breast cancer risk and any R462Q genotype [29]. Recently, a strong link was established between impaired RNase L activity and infection by a novel gammaretrovirus [30]. cDNA from prostate cancer patients with different genotypes for R462Q, were used to screen an oligonucleotide array containing the most conserved sequences from all known viruses. Forty percent of the patients homozygous for 462Q were found to be infected by the xenotrophic murine leukemia virus (XMLV), whereas only 1.5% of patients being heterozygous and homozygous (RR) carried the virus. Cells, homozygous for Q have a decreased RNase L catalytic activity and loose the ability to go into apoptosis, strongly indicating that RNase L plays a role in clearing viral infection.

It is estimated that 80% of all adults will acquire an HPV infection at some point of their life. Worldwide, 500,000 women are diagnosed with cervical cancer and the prevalence of a genital HPV infection is estimated to be 326 million among adult women [31].

HNSCC is the sixth most frequent cancer form with 650,000 new incidents and 350,000 deaths per year worldwide [31]. The major risk factors are tobacco and alcohol consumption, body mass and HPV infection, the latter is especially associated with oropharyngeal cancer [32].

Breast Cancer is the second most frequent cancer in the world and the most common in women with more than 1 million new cases and more than 411,000 deaths per year [33]. The major risk factors are age, early menarche, late age of first pregnancy, late menopause, hormone exposure, and lack of exercise [34], [35].

HPV is the major risk factor for uterine cervical cancer [36] and responsible for approximately one fourth of the HNSCC cases [37]. The highest prevalence is found for squamous cell carcinomas (SCC) located at the tonsils, oropharynx, oral and larynx [38]. The HPV strains 16 and 18 are associated with 99.7% of all cervical cancers [39] and HPV-16 with 86.7% of oropharyngeal SSC, 68.2% of oral SSC and 69.2% of laryngeal SSC [37]. HPV-16 and 18 are among the high-risk viruses, encoding the oncoproteins E6 and E7, which are capable of silencing p53 and pRb, respectively. Thereby, they are omitting important cell cycle checkpoints [40], [41].

Di Lonardo et al. were the first to identify HPV-16 DNA in breast carcinomas [42]. Worldwide studies show a considerable geographic difference in the prevalence of HPV in breast carcinomas varying from 0–86%, described in [43]. In addition, the mouse mammary tumor virus (MMTV) has been found in breast tumors, and insertion sites in the human genome affects genes, which are part of gene families and pathways involved in breast carcinogenesis [44]. Likewise, Epstein Barr virus (EBV), human herpes virus-8 (HHV-8) and bovine leukemia virus (BLV) have been detected in breast tumors, reviewed in [45].

The majority of viral infections are cleared by the immune system, and a genetic predisposition could explain why the infection persists and induce cancer in some individuals.

The aim of this study was to establish a specific genotype or a combination of genotypes (haplotype) across *RNASEL*, which could prove to be a marker for the individual risk of acquiring a persistent HPV infection, thereby an increasing risk of developing especially uterine cervix and head and neck cancer. The promoter region and the 5′ and 3′ UTR were included in the search for new mutations. A yet uncharacterized 5′UTR mutation, being in linkage disequilibrium (LD) with well characterized missense mutations in exon 2, proved to be a statistical strong marker for increased risk and heterozygous protection from all three cancer forms.

## Results and Discussion

### Mutational spectrum of *RNASEL* in cervical carcinomas

As viral infection is implicated in close to 100% of all uterine cervix cancer cases, we hypothesized that if a defective RNase L is responsible for the lack of ability to clear a viral infection, patients with cervix cancer would carry a wide spectrum of mutations affecting the activity or expression level of RNase L. Therefore, leukocyte DNA from 42 patients diagnosed with uterine cervix cancer were screened for mutations by direct sequencing of both DNA strands of all coding and regulatory regions of *RNASEL*. The HPV genotype was established on DNA isolated from each tumor.

**Figure 1:**
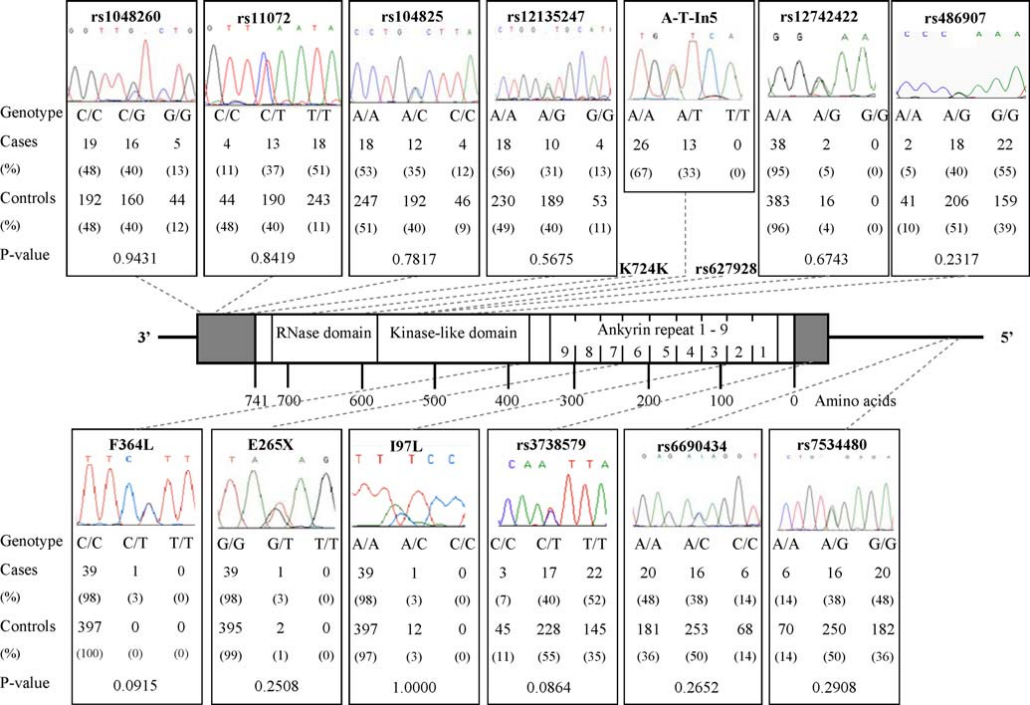
The entire coding and flanking intron sequences, the 5′ and 3′ UTR and the promoter region of *RNASEL* were analyzed for mutations by direct sequencing of leukocyte DNA from patients with cervix cancer (primers are listed in table 3). The two well-described exonic SNPs (rs627928 and K724K) are not illustrated. Genotype frequencies of the SNPs determined in the controls are given beneath the illustration of each SNP, along with a P-value of the marginal effect of the SNP. The Bonferroni corrected significance level is 0.05/12 = 0.004. The genotype frequencies of the cases in the remaining 3 SNPs are: rs627928–7:20:13 (G/G:G/T:T/T); A-T In5–26:13:0 (A/A:A/T:T/T) and K724K–37:3:0 (A/A:A/G:G/G). The ideogram color code is defined as: white, coding region; grey, UTR; black, intragenic regions.

We identified 15 different mutations: four non-synonymous (I97L, F364L, R462Q and D541E), one nonsense (E265X), one synonymous (K724K) and nine in un-translated regions (Figure 1).

The mutations of I97L and E265X are positioned in the ankyrin repeat three and seven, respectively, and F364L, R462Q and D541E are part of the kinase-like domain. No mis- or nonsense mutations were found in the RNase domain in consistency with previous reports (reviewed in [46], [47]). One highly polymorphic T to C mutation (rs3738579) was present in 5′UTR of exon 2, 95 bp upstream from the translation initiation codon. The intron mutations comprised rs12742422, an A>G, 59 bp from the 5′ splice site in intron 2 and A>T In5, an A to T 34 bp from the 3′ splice site in intron 5. The four mutations in the 3′UTR comprised rs12135247, rs104825, rs11072 and rs1048260. None of these were located in any of the characterized regulatory sequences [48]. Mutations in the promoter region comprised rs7534480 within the Neurogenin binding site (*NGN1/3*) and rs6690434, a highly conserved residue within the myoblast-determining factor (*MYOD*), the positions given are according to [49].

Non-synonymous and nonsense mutations with the exception of F364L, reported here for the first time, have previously been characterized with specific focus on familial and sporadic prostate cancer. F364L is located on the border of the protein kinase-like domain and no implication of F364L on the activity of RNase L has yet been established. One patient was heterozygous for F364L, whereas none was found among 407 controls in the current study.

None of the cervix cancer patients were homozygous for any of the rare alleles of the intronic mutations (Figure 1). It is not known whether any of these mutations affect the translation or the splicing efficiency.

We established the genotype frequencies of twelve out of the fifteen mutations found in *RNASEL* in a Danish control population (*n = *502) using the primer extension method. The number of successfully genotyped controls varies according to the specific mutation (Figure 1).

### Comparison of genotype frequencies in different cohorts

The genotype frequencies were compared between the cervix cancer patients and the controls using Fisher's exact test (Figure 1). The frequencies of SNP rs3738579 were found to differ most in the two groups (P-value: 0.086), but none of the SNPs showed significant differences. Subsequently, we investigated three SNPs in 199 Danish patients diagnosed with primary unilateral breast cancer and in 382 Danish HNSCC patients. SNP rs3738579 was chosen because it showed the most deviating frequencies; SNP R462Q because it is known to affect the RNase L activity, where the genotypes GA or AA are associated with a decrease in RNase L activity, reduced ability to clear viral infections, linked to familial prostate cancer, and to more advanced disease in pancreatic cancer [13], [27]; SNP rs6690434, which is located in the promoter region of *RNASEL* affecting a potential MyoD binding site, could be of possible regulatory importance. RNase L has been found to regulate MyoD mRNA stability and to be involved in myoblast differentiation [50].

Random samples were sequenced to verify the genotypes, established by the primer extension method, of each SNP (rs3738579, SNP R462Q and rs6690434). In total, samples from all cervix cancer patients, 47% (239/504) controls, 34% (62/185) breast cancer patients and 13% (47/375) HNSCC patients were sequenced to verify the interpretation of the genotypes.

### The implication of the 5′UTR SNP rs3738579 in viral and non-viral induced cancer

The genotype frequencies for each sample of cancer patients (breast and HNSCC) were compared to the controls. A significant difference was seen only for rs3738579 in breast cancer, P-value = 0.0012 and in HNSCC, P-value = 0.0006.

We found that the genotype frequencies for rs3738579 were similar in our control population and the HapMap European (CEU) population [51], as opposed to the genotype frequencies in the three similar cancer cohorts (see Table 1). Interestingly, a combination of data from all three cancer forms provided strong statistical evidence for rs3738579 as a cancer marker (P-value: 4.43×10^−5^). This finding points to *RNASEL* as a general marker for cancer risk being not exclusively linked to viral induced cancer forms. In addition, heterozygous individuals were overrepresented in the controls, implying a heterozygote advantage and potential protection against the three cancer forms.

**Table 1:** Genotype frequencies for SNP rs3738579

Genotype	Control population	HapMap data	Cervix cancer patients	Breast cancer patients	HNSCC patients
CC	45 (0.108)	7 (0.117)	3 (0.071)	34 (0.184)	54 (0.144)
CT	228 (0.545)	33 (0.550)	17 (0.405)	73 (0.395)	153 (0.408)
TT	145 (0.347)	20 (0.333)	22 (0.524)	78 (0.422)	168 (0.448)

The genotype frequencies are given for controls, n = 502, the CEU HapMap population, n = 60 cervix cancer, n = 42, breast cancer, n = 199 and HNSCC, n = 382, respectively, and the proportion of individuals with the genotype is given as percent in brackets. The genotype frequencies of the controls and the HapMap population are similar (P-value: 0.95), and the genotype frequencies of the three cancer populations are similar (P-value for cervix and breast: 0.18; cervix and head and neck: 0.41; breast and head and neck: 0.48).

Considering pairs of SNPs, none of the pairs show an association with any of the three types of cancer, or all jointly, stronger than the marginal association with rs3738579, indicating that rs3738579 is a better marker than any combination of two SNPs.

Table 2 shows odds ratios (ODs) for the three different genotypes of rs3738579; overall the ODs are similar for the three types of cancer.

**Table 2:** Odds Ratio for the SNP rs3738579 in the three cancer samples and in the samples combined, compared to the control population.

Genotype: rs3738579	Cervix Cancer:	Head and Neck Cancer (HNSCC):	Breast Cancer:	Total:
CC	0.638	1.394	1.866	1.476
CT	0.567	0.574	0.543	0.564
TT	2.071	1.528	1.372	1.511

### Linkage disequilibrium (LD) across *RNASEL*

**Figure 2:**
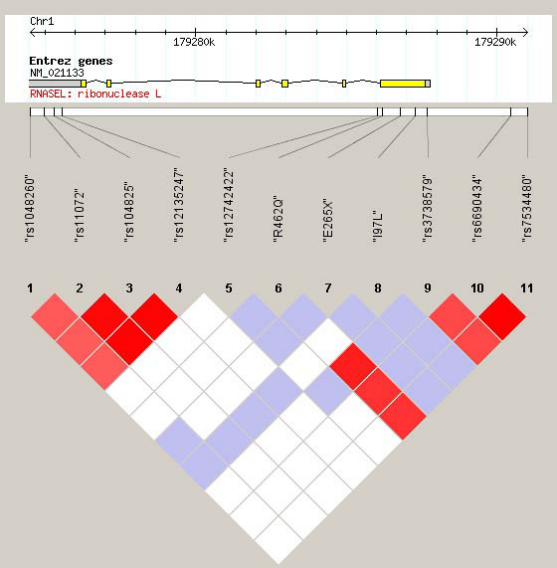
Linkage Disequilibrium (LD)-plot of the region containing *RNASEL.* Each square indicates the level of LD between two SNPs. The color code is defined as: red (high LD), LOD(2 and D′ = 1; shades of pink/red, LOD(2 and D′<1; blue, LOD<2 and D′ = 1; white, LOD<2 and D′<1. The top of the figure shows the genomic position and the known genes in the region (yellow: exon; grey: UTR; black line: intron) [54]. Note that exon 1 is not shown. The two SNPs within the promoter are illustrated to the far right.

To our knowledge the implication of rs3738579 on activity or expression of RNase L is unknown. The mutation is not located within a uORF (un-translated open reading frame) but may change the conformation of the mRNA. One explanation could be that rs3738579 is in linkage disequilibrium (LD) with a not yet identified mutation in the regulatory regions of *RNASEL* or a causative mutation in a nearby gene within the same haploblock (see Figure 2). The LD pattern across the sequenced region for the control population was calculated. Two independent LD blocks were found, one in the 3′UTR region and one in the region spanning the promoter region and exon 2. If a causative mutation exists, being in LD with rs3738579, it can only be present upstream from the introns flanking exon 2, since all exons, flanking intron and promoter sequences have been directly sequenced in all 42 cervical cancer patients.

A heterozygous advantage was found in the study linking infection by a novel gammaretrovirus (XMLV) to the R462Q genotypes. Here, 40% of the analyzed prostate cancer patients, being homozygous for the Q allele, and 1.9% of the patients being homozygous for the R allele, were infected by XMLV. None of the analyzed heterozygous patients carried the infection [30]. This mutation is in LD with rs3738579, where the heterozygous genotypes of both SNPs are linked as well as homozygosity for the rare and frequent alleles, respectively.

### Establishment of the HPV genotype

**Figure 3:**
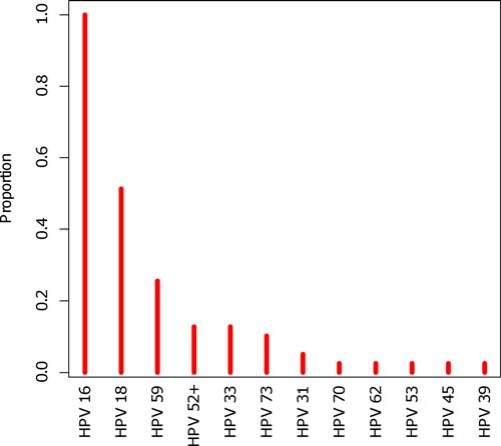
Distribution of HPV genotypes in cervical carcinomas. Thirty-two uterine cervix tumors were infected by more than one HPV strain and all 42 cases were infected by the high-risk HPV16.

The HPV genotype was established in DNA isolated from cervical carcinomas using the Linear Array HPV Genotyping Test kit (Roche Diagnostics GmbH, Mannheim, Germany), which allow for detection of 37 different HPV genotypes including 13 from the high-risk group. We found no correlation between HPV strains and rs3738579 genotypes. All cervical tumor samples were infected with HPV16 and in addition 52% with HPV18, in total eleven different HPV strains were identified in the 42 cervix patients (Figure 3). In comparison, HNSCC is a highly heterogeneous disease with predominantly squamous cell carcinomas and only 30% of the study sample was expected to carry an HPV infection. This strengthens the hypothesis presented here that RNase L may be a general marker for cancer risk and not specifically linked to viral induced cancers.

### Conclusion

A heterozygote advantage and protective potential of *RNASEL* have not previously been reported. SNP rs3738579 is located within the 5′UTR region and may be implicated in posttranscriptional regulation or a mediator of an existing low penetrant mutation. One such mutation could be the R462Q located within the same exon. 462Q decreases the activity of RNase L up to three fold and has been intensively studied in large prostate cancer cohorts, but the results are highly contradictive. Linkage to rs3738579 may help explain these diverse results.

Interestingly, the implication of a mutated *RNASEL* on viral induced cervical cancer has not been described before, despite the well-documented role of the enzyme in the degradation of viral RNA. Furthermore, characterization of the new 5′UTR mutation rs3738579 in patients with sporadic breast and HNSCC led to the hypothesis that RNase L is a general marker for cancer risk, not restricted to familial prostate cancer.

## Materials and Methods

### Cervix cancer patients

Pre-treatment buffy coat blood samples, (n = 40) and tumor tissue samples, (n = 42) were collected from patients with newly diagnosed carcinoma of the uterine cervix. Median age was 59 (range 28–84). Twenty-nine women were postmenopausal, 12 premenopausal. Nineteen women were current tobacco smokers and smoking history was not available in five cases. Tumors were FIGO stage IB, n = 4; IIA, n = 1; IIB, n = 20; IIIa, n = 2 and IIIIB, n = 15. The majority of tumors were squamous cell carcinoma, n = 37 while one was an adenocarcinoma, one mixed adeno/neuroendocrine and 3 were adenosquamous carcinoma.

### DNA preparation from fresh frozen cervix cancer tissue for HPV analysis

DNA was isolated from 42 fresh frozen tumor tissues according to the manufacturers' recommendations for the Linear Array HPV Genotyping Test kit (Roche Diagnostics GmbH, Mannheim, Germany). In brief, the tissue was dissolved in 250 μl PBS, 80 μl tissue lysis buffer (ALT) and 20 μl Proteinase K, mixed/vortexed briefly and incubated for 60 min at 56°C. 250 μl lysis buffer (AL) containing carrier RNA was added, mixed and incubated at 70°C for 30 min. 300 μl 96% ethanol was added, the solutions were mixed thoroughly and incubated for 5 min at room temperature. The tubes were briefly centrifuged and all liquid transferred to a QlAamp column, the lysate was removed by a vacuum pump, 750 μl wash buffer (AW2) was added and left for 1 min. The wash buffer was removed and the samples washed with 750 μl 96% ethanol. The filter from the column was transferred to a new tube. 120 μl elution buffer was added and collected by centrifugation after 2 min incubation at room temperature.

The samples can be stored at 2–8°C for 7 days or at 20°C for 8 weeks.

DNA was purified according to standard procedures.

### Control population

DNA was extracted from peripheral blood according to a modified salt precipitation method from 502 Danish medical students of both sexes in their first year at Medical school [52]. The Local Ethical Committee, Aarhus County, Denmark, approved the current study. All individuals were informed and consented both verbally and in writing.

### Breast cancer patients

Matching blood and tumor samples were collected from 199 primary breast cancer patients. All patients had primary unilateral breast carcinoma without evidence of disseminated disease, no other malignancies. Complete clinical, histopatological and biological information were available, the cohort is described in [53]. Purification of DNA was carried out using a modified salt precipitation protocol as described in [52].

### Head and Neck cancer patients

DNA was derived from fibroblast cultures obtained from 382 consecutive patients with HNSCC treated at the Aarhus University Hospital. The patients were treated with primary radiotherapy according to the protocols and guidelines from the Danish Head and Neck Cancer Group (http://www.dahanca.dk/). The primary tumor sites were larynx, oropharynx, hypopharynx, nasopharynx and oral cavity.

### Ethical approval

The Local Ethical Committee, Aarhus County, Denmark, approved the current study. All patients were informed and consented both verbally and in writing.

### Mutation analysis

Both strands of all DNA fragments were sequenced. Primer sequences and PCR conditions are listed in table 3. The initial standard PCR amplification comprised 20 ng DNA, 1 pmol of each primer, 250 μM dNTP (Roche), 1×buffer (supplied with the enzyme) and 0.5 unit Taq polymerase (Roche) in a final volume of 25 μl.

**Table 3:** Primer sequences for DNA sequencing and SNP genotype assessment

Polymorphism	Oligonucleotide name	Oligonucleotide sequence 5′-3′
rs7534480	SNPrs7534480	GGTGATGGTGCCATCTGT
	*RNASEL*Prr	GTCTCCAAAGCCCAAGAATTC
	*RNASEL*Prf	GATGGTGGGTCAATGATGC
rs6690434	SNPrs6690434	GTGAGTTGAGTGAGCTGAGA
	*RNASEL*Prr	GTCTCCAAAGCCCAAGAATTC
	*RNASEL*Prf	GATGGTGGGTCAATGATGC
rs2274509	SNPrs2274509	CTGGAGGAAAGCAATAGCC
	*RNASEL*Prr	GTCTCCAAAGCCCAAGAATTC
	*RNASEL*Prf	GATGGTGGGTCAATGATGC
rs3738579	SNPT73C	GTTGCCAGAGAATCCCCAA
	*RNASEL*2ar2	CTCATTGACATCTGCTC
	*RNASEL*2prf2	GATTCAAGTGTTTTCTCCC
I97L	SNPI97L	GCAATCGCTGCGAGGA
	*RNASEL*2ar2	CTCATTGACATCTGCTC
	*RNASEL*2af	TGCATTTTCTCAAGGAAAAGGC
E265X	SNPE265X	CTGGAGCAAGAGCACATA
	*RNASEL*2br	GAGGGTGAAAATCTTCTTTGGC
	*RNASEL*2bf3	AGTGGAGAAGAAGCACTTGG
F364L	SNPF364L	GATTGGCAAACTCAAGTTC
	*RNASEL*2cr2	CTCTAGGCCTTTCCTCTC
	*RNASEL*2cf	GGATCTTGTTATGACAGCG
rs486907	SNPR462Q:	AAATATAGATGACAGGACATTT
	*RNASEL*2cr	CTCTAGGCCTTTCCTCTC
	*RNASEL*2cf2:	CAGTCACTTGGTGACATTC
rs627928	SNPD541E:	GAAGCATCTCATTTGAGGA
	*RNASEL*4r	CCCTTTCCATCCTGGAGG
	*RNASEL*	CATCTTTGGCTTGATTTATGG
AGIntron2	SNPAGIn2:	GGGATCTTTGTTTATGATAGG
	*RNASEL*In2r:	GAACCCAGTTATAATTGGTAG
	*RNASEL*2cf2:	CAGTCACTTGGTGACATTC
rs516134	SNPrs516134	GGAA AGGCTGTTCT TGCCT
	rs516134r	GCACAGATTTGTAAAGGAC
	rs516134f	GAGGTCTAAGTGCAATGATG
rs533259	SNPrs533259	CTTGATGCCATCACAGCTTC
	rs533259r	CTGACTGTCCAGCATGAG
	rs533259f	GAACCAGTAAGGGGTGC
ATIn5	SNPATIn5:	CTAAATGATCTAAATGATCATGA
	*RNASEL*5r	GACTAACCCCTGCACTATAGG
	*RNASEL*5f	GGAAAGGGAGGGATGGGATG
rs11807829	SNPK724K	CAGCTCCATCACACTGAGG
	*RNASEL*7r	CTCTACAGCTAATAAGTAGTTC
	*RNASEL*7f	CAAGCATGCTGAACAATTTGTG
rs12135247	SNPrs12135247	GTATAAGTCTGGGCACTGG
	*RNASEL*3′UTRr	TGTACGAAGATGGTTCATTC
	*RNASEL*3′UTRf	CTGGTGATCTATGTCTACAC
A857C, 3′UTR	SNPrs104825	GCTGAGGTTGAGAAAACCUG
	*RNASEL*3′UTRr	TGTACGAAGATGGTTCATTC
	*RNASEL*3′UTRf	CTGGTGATCTATGTCTACAC
rs11072	SNPrs11072	GTCTGCCAAGTGGGAATGTT
	*RNASEL*3′UTRr	TGTACGAAGATGGTTCATTC
	*RNASEL*3′UTRf	CTGGTGATCTATGTCTACAC
rs1048260	SNPrs1048260	GGTGCTCATTACAAATCAGA
	rs1048260r	CAGAAACTCTCAGAGAATTC
	rs1048260f	CTTCCCATACCCAGTCTA

PCR cycle conditions were as follows: 1 cycle of 94°C for 4 min, 35 cycles of 93°C for 35 sec, 59°C for 40 sec, 72°C for 1 min followed by 72°C for 10 min. Products were analyzed by electrophoresis in a 1% agarose gel stained with ethidium bromide. Generation of a single stranded product for sequencing comprised: 2 μl PCR product from above, 5 pmol primer, 6 μl of a 2.5×buffer (supplied with the BigDye kit, ABI, Foster City, CA) and 2 μl Reaction mix (BigDye ver. 2.0 and 1.1; ABI, Foster City, CA) supplied with ddH_2_O to a final volume of 20 μl. Conditions for cycle sequencing were as follows: 1 cycle of 96°C for 5 min, 25 cycles of 96°C for 30 sec, 59°C for 15 sec, 60°C for 4 min. The products were separated by capillary electrophoresis (ABI 3100, Applied Biosystems, Foster City, CA) and evaluated using the software Sequencing analysis® (Applied Biosystems, Foster City, CA).

### Single nucleotide polymorphism (SNP) detection

The primer sequences for PCR amplification of the target region containing one or additional SNPs, and for detecting the individual SNP are listed in Table 3.

The PCR amplification comprised: 20 ng DNA, 200 μM dNTP, 0.4 Units Taq polymerase (Ampliqon, Bie and Berntsen, DK), 1×buffer supplied with the enzyme and additional 1.6 mM MgCl_2_ in a final volume of 15 μl. Depending on the DNA fragment the annealing temperature was ranging between 54–60°C. The genotypes were determined by single nucleotide extension using the SNaPshot® kit following the manufacturers' instructions (Applied Biosystems, Foster City, CA). All products were detected via capillary electrophoresis using the software GeneMapper® (ABI 3100, Biosystems Solutions, Foster City, CA).

### HPV detection

The Linear Array HPV Genotyping Test kit (Roche Diagnostics GmbH, Mannheim, Germany) allows for detection of 37 different HPV genotypes including 13 from the high-risk group. The differentiation between HPV genotypes is based upon PCR amplification of the highly polymorphic L1 region followed by hybridization to specific oligonucleotide probes and colorimetric detection. The manufacturers' guidelines were followed.

### Statistical methods

A Bonferroni corrected Fisher's exact test is used for all tests. For cervical cancer 12 tests are for single marker associations, yielding a Bonferroni corrected significance level of 0.05/12 = 0.004, and 66 tests are made for SNP-pair associations yielding a corrected level of 0.05/66 = 0.0008. For breast cancer and HNSCC 3 tests are made for single marker associations, yielding a Bonferroni corrected significance level of 0.05/3 = 0.0167, and 3 tests are made for SNP-pair associations.

Missing data are handled by omitting individuals with missing data in the genotype counts; e.g. all individuals with missing data in either rs3738579 or R462Q are omitted when calculating the genotype frequencies of that specific SNP-pair.

ODs are calculated as *p*(1−*q*)/[(1−*p*)*q*] where *p* is the frequency of one of the three genotypes in the cancer samples (cervix, head and neck, breast, or combined) and *q* the frequency of the genotype in the control population.

### HapMap population

The genotype frequencies for SNP rs3738579 in the European HapMap population are found by using the CEU population in the HapMap genome browser with HapMap data rel21a on NCBI build 35. [51]

### Linkage disequilibrium

Linkage disequilibrium (LD) is investigated by the program HaploView (v. 3.31) using genome build NCBI35 [54]. Only polymorphic SNPs are shown in Figure 2.

## References

[1] Zhou A, Hassel BA, Silverman RH (1993). Expression cloning of 2-5A-dependent RNAase: a uniquely regulated mediator of interferon action.. Cell.

[2] Hassel BA, Zhou A, Sotomayor C, Maran A, Silverman RH (1993). A dominant negative mutant of 2-5A-dependent RNase suppresses antiproliferative and antiviral effects of interferon.. Embo J.

[3] Xiang Y, Wang Z, Murakami J, Plummer S, Klein EA (2003). Effects of RNase L mutations associated with prostate cancer on apoptosis induced by 2′,5′-oligoadenylates.. Cancer Res.

[4] Rusch L, Zhou A, Silverman RH (2000). Caspase-dependent apoptosis by 2′,5′-oligoadenylate activation of RNase L is enhanced by IFN-beta.. J Interferon Cytokine Res.

[5] Roy FL, Salehzada T, Bisbal C, Dougherty JP, Peltz SW (2005). A newly discovered function for RNase L in regulating translation termination.. Nat Struct Mol Biol.

[6] Hovanessian AG, Brown RE, Kerr IM (1977). Synthesis of low molecular weight inhibitor of protein synthesis with enzyme from interferon-treated cells.. Nature.

[7] Kerr IM, Brown RE (1978). pppA2′p5′A2′p5′A: an inhibitor of protein synthesis synthesized with an enzyme fraction from interferon-treated cells.. Proc Natl Acad Sci U S A.

[8] Naik S, Paranjape JM, Silverman RH (1998). RNase L dimerization in a mammalian two-hybrid system in response to 2′,5′-oligoadenylates.. Nucleic Acids Res.

[9] Dong B, Niwa M, Walter P, Silverman RH (2001). Basis for regulated RNA cleavage by functional analysis of RNase L and Ire1p.. Rna.

[10] Tanaka N, Nakanishi M, Kusakabe Y, Goto Y, Kitade Y (2004). Structural basis for recognition of 2′,5′-linked oligoadenylates by human ribonuclease L.. Embo J.

[11] Manning G, Whyte DB, Martinez R, Hunter T, Sudarsanam S (2002). The protein kinase complement of the human genome.. Science.

[12] Dong B, Silverman RH (1999). Alternative function of a protein kinase homology domain in 2′, 5′-oligoadenylate dependent RNase L.. Nucleic Acids Res.

[13] Casey G, Neville PJ, Plummer SJ, Xiang Y, Krumroy LM (2002). RNASEL Arg462Gln variant is implicated in up to 13% of prostate cancer cases.. Nat Genet.

[14] Liu CY, Wong HN, Schauerte JA, Kaufman RJ (2002). The protein kinase/endoribonuclease IRE1alpha that signals the unfolded protein response has a luminal N-terminal ligand-independent dimerization domain.. J Biol Chem.

[15] Patmore HS, Ashman JN, Cawkwell L, MacDonald A, Stafford ND (2004). Can a genetic signature for metastatic head and neck squamous cell carcinoma be characterised by comparative genomic hybridisation?. Br J Cancer.

[16] Halder A, Halder S, Fauzdar A (2005). A preliminary investigation of genomic screening in cervical carcinoma by comparative genomic hybridization.. Indian J Med Res.

[17] Hoglund M, Gisselsson D, Hansen GB, Sall T, Mitelman F (2002). Multivariate analysis of chromosomal imbalances in breast cancer delineates cytogenetic pathways and reveals complex relationships among imbalances.. Cancer Res.

[18] Carpten J, Nupponen N, Isaacs S, Sood R, Robbins C (2002). Germline mutations in the ribonuclease L gene in families showing linkage with HPC1.. Nat Genet.

[19] Rokman A, Ikonen T, Seppala EH, Nupponen N, Autio V (2002). Germline alterations of the RNASEL gene, a candidate HPC1 gene at 1q25, in patients and families with prostate cancer.. Am J Hum Genet.

[20] Rennert H, Bercovich D, Hubert A, Abeliovich D, Rozovsky U (2002). A novel founder mutation in the RNASEL gene, 471delAAAG, is associated with prostate cancer in Ashkenazi Jews.. Am J Hum Genet.

[21] Li H, Tai BC (2006). RNASEL gene polymorphisms and the risk of prostate cancer: a meta-analysis.. Clin Cancer Res.

[22] Nakazato H, Suzuki K, Matsui H, Ohtake N, Nakata S (2003). Role of genetic polymorphisms of the RNASEL gene on familial prostate cancer risk in a Japanese population.. Br J Cancer.

[23] Wiklund F, Jonsson BA, Brookes AJ, Stromqvist L, Adolfsson J (2004). Genetic analysis of the RNASEL gene in hereditary, familial, and sporadic prostate cancer.. Clin Cancer Res.

[24] Wang L, McDonnell SK, Elkins DA, Slager SL, Christensen E (2002). Analysis of the RNASEL gene in familial and sporadic prostate cancer.. Am J Hum Genet.

[25] Maier C, Haeusler J, Herkommer K, Vesovic Z, Hoegel J (2005). Mutation screening and association study of RNASEL as a prostate cancer susceptibility gene.. Br J Cancer.

[26] Rennert H, Zeigler-Johnson CM, Addya K, Finley MJ, Walker AH (2005). Association of susceptibility alleles in ELAC2/HPC2, RNASEL/HPC1, and MSR1 with prostate cancer severity in European American and African American men.. Cancer Epidemiol Biomarkers Prev.

[27] Bartsch DK, Fendrich V, Slater EP, Sina-Frey M, Rieder H (2005). RNASEL germline variants are associated with pancreatic cancer.. Int J Cancer.

[28] Kruger S, Silber AS, Engel C, Gorgens H, Mangold E (2005). Arg462Gln sequence variation in the prostate-cancer-susceptibility gene RNASEL and age of onset of hereditary non-polyposis colorectal cancer: a case-control study.. Lancet Oncol.

[29] Sevinc A, Yannoukakos D, Konstantopoulou I, Manguoglu E, Luleci G (2004). Lack of association between RNASEL Arg462Gln variant and the risk of breast cancer.. Anticancer Res.

[30] Urisman A, Molinaro RJ, Fischer N, Plummer SJ, Casey G (2006). Identification of a novel Gammaretrovirus in prostate tumors of patients homozygous for R462Q RNASEL variant.. PLoS Pathog.

[31] Parkin DM, Bray F, Ferlay J, Pisani P (2005). Global cancer statistics, 2002.. CA Cancer J Clin.

[32] Ragin CC, Taioli E (2007). Survival of squamous cell carcinoma of the head and neck in relation to human papillomavirus infection: review and meta-analysis.. Int J Cancer.

[33] Kamangar F, Dores GM, Anderson WF (2006). Patterns of cancer incidence, mortality, and prevalence across five continents: defining priorities to reduce cancer disparities in different geographic regions of the world.. J Clin Oncol.

[34] Lilienfeld AM, Johnson EA (1955). The age distribution in female breast and genital cancers.. Cancer.

[35] Henderson BE, Feigelson HS (2000). Hormonal carcinogenesis.. Carcinogenesis.

[36] zur Hausen H (2002). Papillomaviruses and cancer: from basic studies to clinical application.. Nat Rev Cancer.

[37] Kreimer AR, Clifford GM, Boyle P, Franceschi S (2005). Human papillomavirus types in head and neck squamous cell carcinomas worldwide: a systematic review.. Cancer Epidemiol Biomarkers Prev.

[38] Hobbs CG, Sterne JA, Bailey M, Heyderman RS, Birchall MA (2006). Human papillomavirus and head and neck cancer: a systematic review and meta-analysis.. Clin Otolaryngol.

[39] An HJ, Kim KR, Kim IS, Kim DW, Park MH (2004). Prevalence of human papillomavirus DNA in various histological subtypes of cervical adenocarcinoma: a population-based study. Mod Pathol..

[40] Kubbutat MH, Vousden KH (1998). New HPV E6 binding proteins: dangerous liaisons?. Trends Microbiol.

[41] Thomas M, Matlashewski G, Pim D, Banks L (1996). Induction of apoptosis by p53 is independent of its oligomeric state and can be abolished by HPV-18 E6 through ubiquitin mediated degradation.. Oncogene.

[42] Di Lonardo A, Venuti A, Marcante ML (1992). Human papillomavirus in breast cancer.. Breast Cancer Res Treat.

[43] Choi YL, Cho EY, Kim JH, Nam SJ, Oh YL (2008). Detection of Human Papillomavirus DNA by DNA Chip in Breast Carcinomas of Korean Women.. Tumour Biol.

[44] Theodorou V, Kimm MA, Boer M, Wessels L, Theelen W (2007). MMTV insertional mutagenesis identifies genes, gene families and pathways involved in mammary cancer.. Nat Genet.

[45] Lawson JS, Gunzburg WH, Whitaker NJ (2006). Viruses and human breast cancer.. Future Microbiol.

[46] Silverman RH (2003). Implications for RNase L in prostate cancer biology.. Biochemistry.

[47] Liang SL, Quirk D, Zhou A (2006). RNase L: its biological roles and regulation.. IUBMB Life.

[48] Li XL, Andersen JB, Ezelle HJ, Wilson GM, Hassel BA (2007). Post-transcriptional regulation of RNase-L expression is mediated by the 3′-untranslated region of its mRNA.. J Biol Chem.

[49] Zhou A, Molinaro RJ, Malathi K, Silverman RH (2005). Mapping of the human RNASEL promoter and expression in cancer and normal cells.. J Interferon Cytokine Res.

[50] Bisbal C, Silhol M, Laubenthal H, Kaluza T, Carnac G (2000). The 2′-5′ oligoadenylate/RNase L/RNase L inhibitor pathway regulates both MyoD mRNA stability and muscle cell differentiation.. Mol Cell Biol.

[51] (2003). The International HapMap Project.. Nature.

[52] Hansen LL, Andersen J, Overgaard J, Kruse TA (1998). Molecular genetic analysis of easily accessible breast tumour DNA, purified from the leftover from hormone receptor measurement.. APMIS.

[53] Hansen LL, Yilmaz M, Overgaard J, Andersen J, Kruse TA (1998). Allelic loss of 16q23.2–24.2 is an independent marker of good prognosis in primary breast cancer.. Cancer Res.

[54] Barrett JC, Fry B, Maller J, Daly MJ (2005). Haploview: analysis and visualization of LD and haplotype maps.. Bioinformatics.

